# Health Tract, No. 206

**Published:** 1865

**Authors:** 


HEALTH TRACT, No. 206.

### FOOD AND HEALTH.

BREAD-CRUST baked in an oven until it is very brown, but not black, and then pounded to the fineness of ground coffee, is a safer, cheaper, and quite as agreeable and healthful a substitute for coffee as any other MIXTURE now in use.

Men who have half a dozen irons in the fire are not the ones to go crazy. It is the man of voluntary or compelled leisure who mopes, and pines, and lounges about, who thinks himself into the mad-house or a premature grave. Motion is all Nature's law. Action is the mental and physical salvation of man.

White beans are the cheapest and most nutritious food which can be eaten. Beans and pork furnish nearly all the elements necessary to human subsistence. A quart of beans at eight cents and a pound of pork at twelve cents will feed a small family for a day. Four quarts of beans and two pounds of corned beef, boiled to rags, in fifty quarts of water, will furnish a good meal for forty men, or one and a quarter cents a meal.

SMALL POX.—It is said that as soon as any eruption appears on the skin it is small pox, if, on pressure with the end of the finger, there is the feeling as if a small fine shot had been placed under the cuticle of the skin.

FACE protection from cold.—An ordinary fine wire-gauze mask, such as is sometimes used at masquerades, will keep the face comfortable, even if a fierce wind is blowing, while the thermometer is below zero; a thin vail or a silk handkerchief is a good substitute.

COAL-GAS.—Two young girls were recently found dead in their bed, having retired in perfect health, in consequence of filling a pot with the live coals of a wood-fire, and placing it in the middle of their chamber, with closed doors and windows, the night being very cold. On New-Year's eve of eighteen hundred and sixty-four, Mr. I. F. Hall, aged fifty years, a gentleman of great moral worth, an exemplary citizen and loving father, retired to his chamber in perfect health, but in the morning was found to have been dead several hours; the gas in the room not having been turned fully off, or having been left burning a little, was puffed out by the wind. Within a year a clergyman from the West was found nearly dead in his chamber in New-York. Being unacquainted with the nature of coal-gas, he had blown out the light instead of turning it off. Every chamber ought to have a ventilator out of reach, or, which would be more certain, an open fireplace, which could not easily be closed. Breathing a vitiated atmosphere during sleeping hours, which is nearly one third of a man's entire existence, is sapping the constitution of multitudes. No one ought to be allowed to sleep in a close room. It will destroy the health sooner or later, and inevitably.

